# Antipsychotic Treatment Failure: A Systematic Review on Risk Factors and Interventions for Treatment Adherence in Psychosis

**DOI:** 10.3389/fnins.2020.531763

**Published:** 2020-10-09

**Authors:** Kawtar El Abdellati, Livia De Picker, Manuel Morrens

**Affiliations:** ^1^Collaborative Antwerp Psychiatric Research Institute, University of Antwerp, Antwerp, Belgium; ^2^Scientific Institute for Neuropsychiatric and Psychopharmacological Studies (SINAPS), University Psychiatric Centre Duffel, Duffel, Belgium

**Keywords:** adherence, non-adherence, compliance, antipsychotic, psychosis, schizophrenia, therapeutic drug monitoring

## Abstract

**Objective:** Antipsychotic medication non-adherence has detrimental effects on patients' clinical outcome. It is unclear which risk factors affect adherence most and which interventions are effective at improving adherence to antipsychotic medication. The aim of this systematic review is to summarize evidence exploring risk factors of non-adherence to antipsychotic treatment and effectiveness of intervention to improve adherence in patients with psychotic spectrum disorders.

**Methods:** We conducted a systematic search in PubMed from 1994 to 2019 using a structured search strategy. Studies were quality assessed, and studies reporting on possible risk factors and intervention strategies were synthesized.

**Results:** We reviewed 26 studies on factors related to antipsychotic medication adherence and 17 studies on interventions to improve adherence in patients with psychosis spectrum disorders. Risk factors of non-adherence included younger age, poor illness insight, cannabis abuse, and the presence of severe positive symptoms. Antipsychotic medication adherence was associated with positive attitude toward medication of both patients and their family, family involvement, and illness insight. Somewhat consistent evidence was found for interventions involving family and technology-based interventions and strategies combining depot medication with psychoeducation. However, given the wide range of heterogeneous interventions and methodological limitations, findings must be interpreted with caution.

**Conclusion:** Despite much effort invested in the research area of antipsychotic medication adherence, the heterogeneity in study design and outcome, adding to confounding effects and possible biases, and methodological restraints complicate comparability of the results. Future research in this field should therefore be conducted on patient-tailored interventions, considering risk factors affecting the patient and implementing well-validated, standardized assessment methods. Accordingly, this systematic review seeks to facilitate endeavors improving adherence to antipsychotic treatment by identifying modifiable and non-modifiable risk factors, outlining effective intervention strategies, and proposing recommendations to enhance adherence strategies.

## Introduction

**Table:** 

Treatment resistance	Kane et al. (1988): (1) a minimum of three treatment periods in the preceding 5 years with antipsychotics (from at least two difference chemical classes) at dosages ≥1,000 mg/day chlorpromazine for a period of 6 weeks, each without significant symptomatic relief and (2) no period of good functioning within the preceding 5 years. Kane et al. (2019): failure to respond on any two antipsychotic medications, each at an adequate dose (i.e., equivalent to ≥600 mg/day chlorpromazine) and treatment duration + objective symptom measurements should be used to assess treatment response and medication adherence.
Pseudo-resistance	Lack of response to antipsychotic treatment not attributed to pharmacological inefficiency of the compound but depending on modifiable and non-modifiable factors such as non-adherence (de Bartolomeis et al., 2018)
Non-adherence	Only some or none of the prescribed medication is taken (Kane et al., 2019)

Psychotic disorders are severe mental disorders that are characterized by episodic or long-term dysfunctions of perceptual, cognitive, and emotional processes that cause severe impairments with regard to social and occupational functioning (Howes et al., 2012). A proportion of patients exhibit little clinical response despite treatment with multiple different antipsychotic drugs (Howes et al., 2017), implicating that therapeutic assistance is often challenging with results that are incomplete and unsatisfactory. This therapeutic failure may be partially or completely due to various factors, including not only treatment resistance, regimen appropriateness, and drug tolerability (Lindenmayer et al., 2009) but also adherence to prescribed treatment (Garcia et al., 2016; Howes et al., 2017). Approximately 30% of patients with schizophrenia and related disorders obtain little benefit from standard antipsychotic treatment and are considered to have a treatment-resistant illness profile (Conley and Buchanan, 1997; Meltzer, 1997; National Collaborating Centre for Mental Health, 2009; Lally et al., 2016; Wimberley et al., 2016; Demjaha et al., 2017). Pioneering work by Kane et al. (1988) initiated a chain of works on treatment resistance in schizophrenia, and accordingly, the topic has been discussed at length [see Howes et al. (2017), Kane et al. (2019)]. Notwithstanding, defining treatment resistance and deriving pragmatic recommendations for clinical practice remains problematic. Current guidelines broadly agree in terms of their definition of treatment, with key criteria that include no significant improvement in psychotic symptomatology after treatment with at least two different non-clozapine antipsychotics at adequate dose and duration of time. However, recommendations and clinical outcomes used to evaluate the level of treatment response vary among the guidelines, which is further complicated by the already heterogeneous psychotic patient population (Kane et al., 2019; Barnes et al., 2020), such that substantially inconsistent results can be found across the studies involving these patients (Suzuki et al., 2012).

Another issue in determining treatment response is the concept of pseudo-resistance (Howes et al., 2017), which postulate that certain components can make it appear as if a patient is non-responsive while in reality treatment response can be altered, i.e., through improvement of adherence behavior (de Bartolomeis et al., 2018). Indeed, at least a third of the patients thought to have a treatment-resistant profile have shown to have subtherapeutic plasma antipsychotic levels due to pharmacokinetic factors or to poor adherence (McCutcheon et al., 2015, 2018). Additionally, antipsychotic treatment non-adherence has been identified as one of the main causes for antipsychotic treatment failure (Goff et al., 2010). Although medication non-adherence is a common problem throughout medicine, several factors make it especially challenging in treating patients with psychotic disorders: direct impact of symptoms on cognitive functions (El-Missiry et al., 2015; MacKenzie et al., 2018), lack of illness insight, stigma, comorbid substance abuse, and social isolation (Haddad et al., 2014). Astoundingly, while the number of patients taking antipsychotics has increased over the years, little progress has been made with regard to improving medication adherence in these patients, possibly because the choice of measurement of adherence is a long-standing methodological problem. Measures of medication adherence can be classified in (1) objective indicators of medication intake, such as pills counts, electronic monitoring, and serum or plasma levels of antipsychotics and (2) subjective measures of medication use via patient report or interviewer ratings. Adherence is an observable, measurable behavior and is often reported as a dichotomous variable (adherence vs. non-adherence), while it can vary along a continuum in which absolute adherence and non-adherence are the two ends. However, the absence of consensus on cutoff points prevents comparability of the literature (Sendt et al., 2015). Although continuous observation of actual medication intake is the true gold standard of adherence estimation, such conspicuous monitoring would prompt better adherence than would occur in unobserved environments. Nonetheless, measuring adherence behavior does not reveal underlying reasons for non-adherence (Sajatovic et al., 2010).

Adherence difficulties complicate the clinical management for prescribers as well. Psychiatrists may have trouble distinguishing between poor adherence and poor treatment response, especially since partial non-adherence occurs as frequently as complete medication cessation (Svestka and Bitter, 2007). A 15-year Belgian population-based study reported that a vast majority of antipsychotic-treated patients took their prescribed medication for a brief period of time (81.8% of the prescribed antipsychotics were administered for a maximum of 3 months), indicating that a considerable part of the patients with psychosis are inadequately or even untreated (Morrens et al., 2015). By underestimating non-adherence, prescribers may prematurely discontinue treatment, add concomitant medications, or increase dosages. Treatment failure in covert non-adherent individuals may lead to the faulty assumption of treatment resistance (Velligan et al., 2013). Clearly, vigorous efforts should be made to determine medication adherence and exclude so-called pseudo-resistant individuals (Howes et al., 2017) in order to improve clinician's decision-making process and prevent further iatrogenic harm (Lopez et al., 2017). In this regard, one could wonder if the routine blood level monitoring for antipsychotics may thus contribute to its superior effectiveness in previously non-responsive patients (Patteet et al., 2012). Moreover, non-adherence has been significantly associated with poorer clinical outcome, including greater risk of hospitalization, longer duration of hospitalization (Higashi et al., 2013; Olivares et al., 2013), and greater risk of suicide (Leucht and Heres, 2006; Llorca, 2008; Forsman et al., 2019). In addition, partial and total medication non-adherence are strongly associated with psychotic relapse as non-adherent patients with schizophrenia having a 5-fold increase in risk of relapse (Robinson et al., 1999; Caseiro et al., 2012). This systematic review will therefore summarize key factors predicting non-adherence in psychotic spectrum disorders (PSDs) in order to better identify at-risk patients. In addition, we evaluate the existing evidence on the efficacy of interventions to improve medication adherence in PSD and their effect on other patient outcomes. To our knowledge, this is the first systematic review combining and linking risk factors and interventions of (non)adherence in psychosis.

## Methods

In August 2019, an electronic search was conducted in the PubMed database for English-language publications from January 1994 to August 2019, using the following MeSH terms: medication adherence, medication compliance, antipsychotics, antipsychotic agents, psychosis, and psychotic disorder. Additionally, we used the following PubMed filters: study type (clinical trials, meta-analysis, observational study, randomized controlled trial, systematic reviews) and study subject (human). Subsequently, reference lists from studies included in our systematic review were manually searched for additional relevant publications. Year 1994 was selected as the start date for the search because of the publication of the Diagnostic and Statistical Manual of Mental Disorders, fourth edition (DSM-IV) in that year.

All abstracts were screened for the following predefined inclusion criteria: clinical trials, observational studies, randomized controlled trials, systematic reviews, and meta-analyses in which the study population consisted of patients with psychosis and schizophrenia spectrum disorders (corresponding to schizophrenia, schizophreniform disorder, schizoaffective disorder, delusional disorder, brief psychotic disorder, other specified schizophrenia spectrum and other psychotic disorder. and unspecified schizophrenia spectrum and other psychotic disorder as described in DSM-V) being treated with antipsychotic agents and in whom factors or interventions associated with treatment adherence were assessed. All studies must include direct and/or indirect measures of medication adherence behavior. Exclusion criteria were other primary diagnosis and narrative or qualitative reviews. To facilitate interpretation of the studies published to date, we considered the distinction between adherence behavior and attitude and excluded studies with an adherence assessment based on adherence attitudes solely.

Quality and risk of bias of the articles related to the objective of our review were assessed using the Critical Appraisal Skills Programme (CASP) Appraisal Checklist (Critical Appraisal Skills Programme, 2019) and the Cochrane risk of bias for randomized studies (Higgins et al., 2011).

### Data Extraction

Two independent reviewers, KEA and LJDP, extracted predefined data and checked the data extraction sheet. Discordant results were resolved through discussion. We developed a standardized data extraction sheet regarding interventions with following data: intervention type, methodology, diagnosis, age, ethnicity, type of antipsychotic, duration, number of included cases, adherence outcome and effects, other outcome measures and effect, definition of (non)adherence, classification of adherence, quantification of adherence, and limitations of the study. A data extraction sheet regarding risk factors and predictors with following data was also created: type of factor, diagnosis, stage of illness, age, ethnicity, type of antipsychotic, methodology, duration of study, number of cases, outcome measures and effect, definition of (non)adherence, and classification and quantification of adherence.

## Results

**Figure 1:**
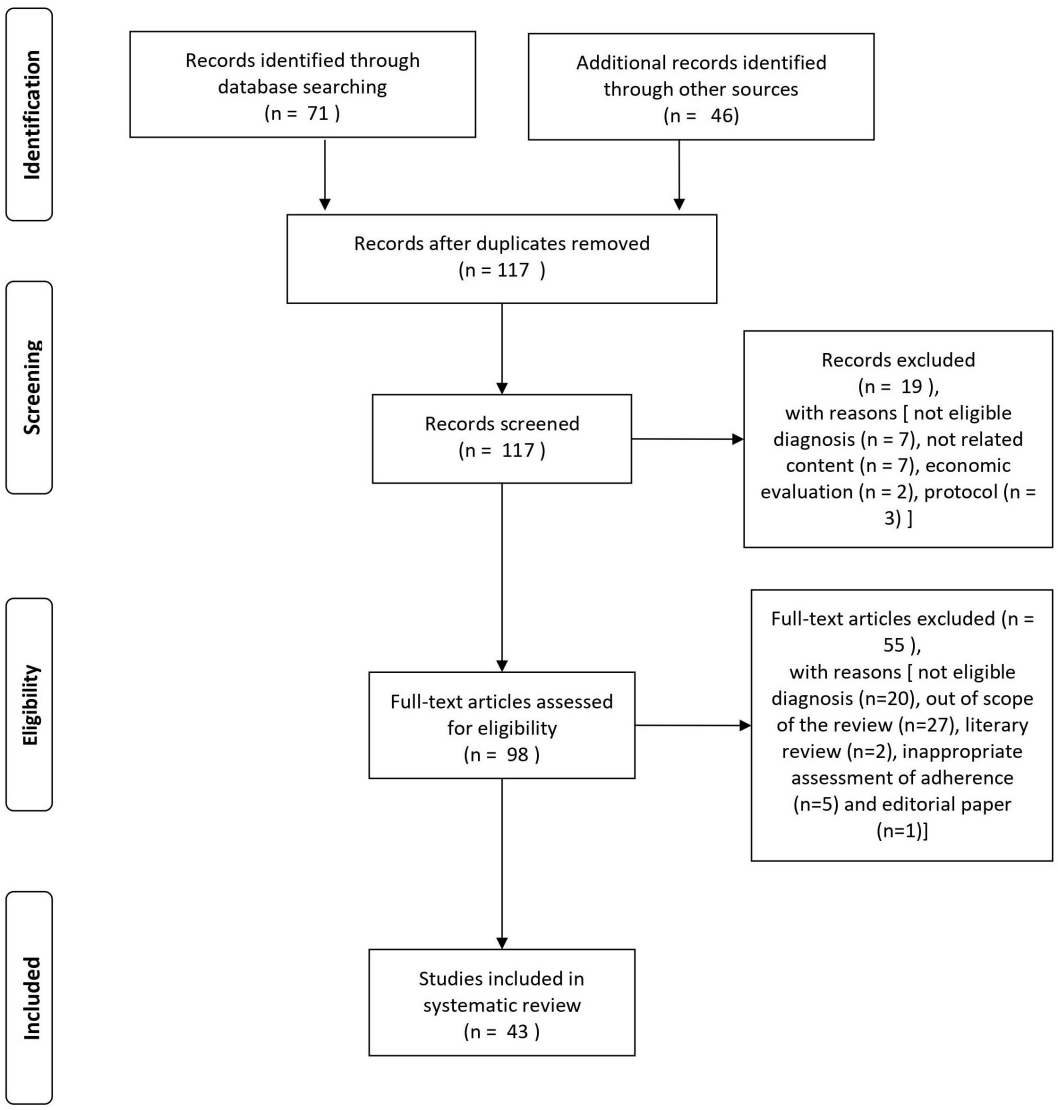
Flowchart of study selection process.

The search of the PubMed database resulted in an initial 71 records (cf. PRISMA flowchart in Figure 1). For three records, we contacted the study authors in order to obtain more information on the characteristics of the study population or for clarification of the results. One of these could provide the necessary information (Beebe et al., 2017). An additional 46 eligible articles were identified by hand search of reference lists. Nineteen articles were excluded at screening with the following reasons: not eligible diagnosis (*n* = 7), not related content (*n* = 7), economic evaluation (*n* = 2), and protocol (*n* = 3). After full-text assessment, additional 55 articles were excluded [not eligible diagnosis (*n* = 20), out of scope of the review (*n* = 27), literary review (*n* = 2), inappropriate assessment of adherence (*n* = 5), and editorial paper (*n* = 1)].

A total of 43 studies was found eligible for the systematic review: 17 studies provided information on intervention strategies to improve antipsychotic medication adherence, and 26 studies were on factors influencing adherence outcome. For a schematic representation of the study selection process, see Figure 1.

### Study Specific Characteristics

Most studies included an adult population, with the exception of one study with an age range of 14–19 years (Molteni et al., 2014). Several studies on the factors associated with medication adherence enrolled participants at early stage of illness (first episode of psychosis, recent onset of psychosis) (Coldham et al., 2002; Mutsatsa et al., 2003; Kahn et al., 2008; Quach et al., 2009; Weiden et al., 2012; Molteni et al., 2014; Winton-Brown et al., 2017). Not all studies reported ethnic background. In general, medication was either taken orally, by depot injection, or in combination. Some studies did not detail specific medication information, reporting them only as antipsychotic or neuroleptic medication.

#### Risk Factors and Predictors of Adherence

The main factors that might influence treatment non-adherence were associated with patients themselves, their drug treatment, and family involvement.

#### Patient-Related Risk Factors and Predictors

Twenty individual studies and three systematic reviews investigated patient-related predictors of non-adherence. The details on each individual study are summarized in Table 1.Sociodemographic features, clinical symptoms, adverse effects, cognitive functioning, illness insight, alcohol and illicit substance use, and patient attitudes are the main factors that have been studied in the context of antipsychotic medication adherence (see Table 2). For an overview of risk factors and predictors related to antipsychotic medication adherence and non-adherence, see Table 3.

**Table 1:** Summary of the characteristics of the individual studies on potential risk factors of adherence and non-adherence.

**Study type**	**Study**	**Sample characteristics**	**Cases**	**Duration of study**	**Adherence measure**	**Adherence rate**	**RoB**
RCT	1. Olivares et al. (2013)	Chronic; stable; SZ + SZA;	599 (10 vs. 20 vs. 40 mg/day, pooled)	8 w (4w and 8 w assessment); DB	Pill counts, response rate, plasma levels for half of patient population	Adherent: 65.5%; non-adherent: 34.5%	Low
	2. Morken et al. (2007)	Recent-onset; stable; SZ + SPH;	30 intervention vs. 20 TAU	24 m (assessments every 2 months); SB	Clinician-rated 4-point scale (based on patient interviews and other measures), family/caregiver reports; plasma levels of AP	Non-adherent: 20%	Low
	3. Weiden et al. (2012)	FEP, acute; PSD	26 intervention vs. 11 TAU	104 w; open-label; SB	Time to initial non-adherence	Non-adherent: 81%	Moderate
	4. Kahn et al. (2008)	FEP; state NR; PSD	Haloperidol (*n* = 103) vs. SGA [amisulpride (*n* = 104), olanzapine (*n* = 105), quetiapine (*n* = 104), ziprasidone (*n* = 82)]	12 m; open-label; unblinded	One-item 7-points rating scale	Non-adherent: haloperidol, 72%; amisulpride, 40%; olanzapine, 33%; quetiapine, 53%; ziprasidone, 45%	Low
CT—open label, naturalistic, flexible-dose	5. Guo et al. (2011)	Early-stage; stable; SZ + SPH	1,133	12 m	Treatment discontinuation rate, including non-adherence or changing initial AP	Non-adherent: chlorpromazine, 41.4%; sulpiride, 39.5%; clozapine, 36.7%; risperidone, 40.2%; olanzapine, 39.6%; quetiapine, 46.9%; aripiprazole, 40.2%	Low
CT—observational, longitudinal	6. Winton-Brown et al. (2017)	FEP; state NR; PSD	136	18 m; retrospective	Self-report, breaks in treatment	Non-adherent: 40.2%	Low
	7. Coldham et al. (2002)	FEP; state NR; PSD	186	3 y (3-monthly assessment 1st year, half-yearly in 2nd year and then annually); prospective	3-point scale	Adherent: 40.9%; inadequately adherent: 19.9%; non-adherent: 39.3%	Low
	8. Mohamed et al. (2009)	Chronic; stable; SZ	1,432	18 m (3-monthly assessment); prospective	Patient, clinician, and family reports; pill counts	Adherent: ±75%	Low
	9. Quach et al. (2009)	FEP; state NR; PSD	547	2 y (annual assessment); prospective	Observer-rated (based on structured interviews with the patient, information from the primary case manager, the psychiatrist, and by systematic examination of the case notes and prescription cards)	Non-adherent: 35–39%	Moderate
	10. Baloush-Kleinman et al. (2011)	Early stage; state NR; SZ + SZA	112	6 m (assessments at admission, discharge, 3 and 6 m FU); prospective	Visual analog scale for assessing treatment adherence (Smith et al., 1992), and rated by patients, relatives, and treating clinician.	Non-adherent: 29.7%	Low
	11. Janssen et al. (2006)	Mixed (10.1% FEP); state NR; PSD	670	Assessment weekly during the inpatient stay (mean stay 43 days), and at discharge; prospective	Likert-type scale within a structured interview, adapted from Amador et al. (1993).	Adherent: 47.0%	Moderate
	12. Acosta et al. (2009)	Mixed (% FEP NR); stable; SZ	74	3 m; prospective	MEMS device, depot visits; estimation by psychiatrist, patients, and family/caregiver reports	Non-adherent: 42.3%	Low
	13. Yang et al. (2012)	Chronic; stable; SZ	65	8 w (assessments at baseline, w 4 and w 8); prospective	MEMS; Pill count; clinician-rated 7-point adherence scale (based on patient interview), patient self-report scale (0–100%)	Non-adherent: 41.2% (MEMS), 7.8% (pill counting), 7.8 %(clinician rating scale), 25.5%'(self-report)	Low
CT—cross-sectional	14. Klingberg et al. (2008)	Mixed (30.6% FEP); stable; SZ + SZA	108	NA	CRS; AP plasma levels	Non-adherent: 0.9%	Low
	15. Mutsatsa et al. (2003)	FEP; acute; SZ + SPH	101	NA	CRS	Non-adherent: 44%	Low
	16. Bayle et al. (2015)	Mixed (% FEP NR); stable; PSD	1,887	NA	MAQ	Non-adherent: 53.2%; partially adherent: 29.5%,; adherent: 17.3%	Low
	17. Molteni et al. (2014)	Early onset (14–19 years); stable; PSD	67	NA	4-point Likert-type questionnaire	Non-adherent: 8.96%; partially adherent: 25.73%; adherent: 65.67%	Low
	18. Day et al. (2005)	Mixed (%FEP NR); acute; SZ + SZA	228	NA	Morisky, DAI	NR	Low
	19. Meier et al. (2010)	Chronic; stable; SZ	409	NA	MAQ, CRS	NR	Low
	20. Borras et al. (2007)	Chronic, stable; PSD	103	NA	Self-report, blood drug monitoring	Non-adherent: 15.5%	Moderate
	21. Aldebot and de Mamani (2009)	Mixed (% FEP NR); stable; SZ + SZA;	40	NA	Modified subscales of the COPE inventory; MARS	NR	Low
	22. McCabe et al. (2012)	Chronic; stable; PSD	507	NA	Clinician-rated: 3-point Buchanan criteria (based on routine clinical contact); for 29% of sample: information from social contacts used to complement clinician rating; objective measures for 49% of sample: depot records, supervised medication taking or drug testing used to inform rating	Poor adherence (<25%): 4.1%; good adherence (>75%): 75.7%	Low
	23. (Jonsdottir et al., 2013)	Illness stage NR; stable; PSD	154	NA	Self-report (Likert 0–100%) + serum concentration (AP in 94.8% of patients)	Full adherence (100% self-report, serum concentration within reference level): 55.2%; no adherence (<12% adherence self-report, no detectable levels): 11.0%; partial adherence (12–95% self-report, detectable serum levels not within reference levels): 51.3%	Low

**Table 2:** Evidence table on risk-factors of antipsychotic medication adherence and non-adherence.

**Study type**	**Study**	**Outcome measures**	**Sociodemographic factors**	**Clinical factors**	**Treatment-related factors**	**Family involvement and therapeutic relations**
RCT	1. Lindenmayer et al. (2009)	PANSS; MADRS; GAF; CGI-S; QLS; Simpson–Angus Scale; BARS and AIMS	Demographics (gender, age, ethnicity), illness characteristics, baseline weight (n.s.)	MADRS scores [baseline total mean (SD), adherent 13.90 (8.80) vs. non-adherent 15.85 (8.50), *p* = 0.010]; worsening PANSS depressive factor (HR = 1.2, 95% CI 1.06–1.35, *p* = 0.003); hostility (HR = 1.14, 95% CI 1.02–1.26, *p* = 0.020); change in PANSS total score and history of substance abuse (n.s.)	adverse events (n.s.) and weight change (n.s.)	
	2. Morken et al. (2007)	Expressed emotion assessment based on CFI	male sex (OR = 6.11, 95% CI 1.2–29.74, *p* = 0.025)	Symptom severity (BPRS) (OR = 1.13, 95% 1.01–1.27, *p* = 0.034)		Patients living with family with high expressed emotion (OR = 36.43, 95% CI 2.18–608.01, *p* = 0.012); lower expressed emotion: 1st year (OR = 19.59, 95% CI 1.64–234.22, *p* = 0.019); both years (OR = 6.04, 95% CI 1.07–34.13, *p* = 0.042)
	3. Weiden et al. (2012)				Route of administration (n.s.)	
	4. Kahn et al. (2008)				FGA vs. SGA (n.s.)	
CT—open label, naturalistic, flexible dose	5. Guo et al. (2011)				FGA vs. SGA (n.s.)	
CT—observational, longitudinal	6. Winton-Brown et al. (2017)	GAF, PANSS, CDSS, insight rating scale (David et al., 1992), relapse	Non-Caucasian (OR = 3, 95% CI 1.3–7.2, *p* = 0.01)	Use of illicit substances (OR = 0.3, 95% CI 0.1–0.5, *p* < 0.001)	Presence of EPS (OR = 8.1, 95% CI 1–65.3, *p* = 0.050)	Carer involvement (OR = 2.2, 95% CI 1–4.9, *p* = 0.048);
	7. Coldham et al. (2002)	QLS; ESRS; Bares Akathisia Scale; Premorbid Adjustment Scale	Young age (*F* = 4.5, *p* = 0.010); young age of onset (*F* = 6.7, *p* = 0.002); younger age (OR = 1.13, 95% CI 1.02–1.24, p = 0.015)	Relapse in first year (*F* = 4.16, *p* = 0.020); positive symptoms at 1 year (*F* = 7.88, *p* = 0.001); QoL at baseline (*F* = 3.45, *p* = 0.030); QoL at 1 y (*F* = 4.47, *p* = 0.010); poor premorbid functioning (OR = 0.07, 95% CI 0.00–0.24, *p* = 0.006); alcohol at baseline (*F* = 3.31, *p* = 0.020); alcohol at 1 y (*F* = 6.21, *p* = 0.003); cannabis at baseline (*F* = 3.17, p = 0.040); cannabis at 1 y (*F* = 3.17, *p* = 0.001); cannabis use (OR = 0.46, 95% CI 0.25–0.84, *p* = 0.012); alcohol abuse n.s.; insight at baseline (*F* = 4.08, *p* = 0.020); insight at 1 y (*F* = 4.26, *p* = 0.02)		lack of family involvement (OR = 0.19, 95%CI 0.05–0.75, *p* = 0.017)
	8. Mohamed et al. (2009)	GAF; ITAQ; DAI		Baseline illness insight (*t* = 2.48, *p* < 0.050); change in insight scores from baseline to follow-up up (ITAQ: 0.078, *p* < 0.001; DAI: 0.235, *p* < 0.001); positive attitudes toward medication (*r* = 0.154, *p* < 0.001)		
	9. Quach et al. (2009)	GAF; SUMD; ROMI	Young age (OR = 1.79, 95% CI 1.16–2.75, *p* = 0.008)	Comorbid addiction (OR = 2.03, 95% CI 1.17–3.52); high global functioning (GAF) (OR = 1.73, 95% CI 1.07–2.81, *p* = 0.0300); unawareness of the effect of medication (OR = 2.34, 95% CI 1.44–3.82, *p* = 0.0010); negative attitude toward medication (OR = 2.13, 95% CI 1.43–3.17, *p* = 0.0001)		No upbringing by both parents (OR = 1.64 95% CI 1.11–2.42, *p* = 0.010); no key supporting relative (OR = 1.54, 95% CI 1.05–2.25, *p* = 0.030)
	10. Baloush-Kleinman et al. (2011)	CGI, SAPS; SANS; Cognitive Appraisal of Health Scale; Scale to Assess Unawareness of Mental Disorder; MacArthur Competence Assessment Tool; ESRS; Liverpool University Neuroleptic Side Effect Rating Scale; patient-rated Trust in Physician Scale; DAI; Visual Analog Scale (perception of family involvement)	Mode of admission, diagnosis of schizoaffective disorder, duration of illness (all n.s.)	Higher levels of insight into illness (*t* = 0.13, *p* = 0.009), awareness of the need for treatment (*t* = 3.82, *p* < 0.001), awareness of the social consequences of illness (n.s.)	Side-effects in adherent group (*t* = 2, *p* = 0.036); medication class (n.s.)	Perceptions of doctor–patient trust in the therapeutic alliance (*t* = 3, *p* = 0.012), perceived family involvement and attitudes toward medication in the family (*t* = 5, *p* < 0.001)
	11. Janssen et al. (2006)	GAF; DOTES; PANSS	Number of previous psychiatric hospitalizations (*p* < 0.010); involuntary admission (OR = 0.60, 95% CI 0.41–0.89, *p* < 0.050); no school graduation (OR = 0.59, 95% CI 0.41–0.86, *p* < 0.010); gender, primary diagnosis, first or multiple episode admission, duration of illness (all n.s.)	History of aggressive behavior (OR = 0.57, 95% CI 0.38–0.85), PANSS negative subscore above 25 (admission) (OR = 0.61, 95% CI 0.43–0.85, *p* < 0.01), PANSS paranoid/belligerence subscore above 9 (admission) (OR = 0.69, 95% CI 0.48–0.99, *p* < 0.01); substance disorder (OR = 0.52, 95% CI 0.32–0.85, *p* < 0.01)	Neurological side effects (n.s.); SGA monopharmacy at discharge > FGA mono or FGA + SGA (*p* < 0.005, *χ*^2^ = 17.6); FGA monotherapy switch to SGA vs. continue to take FGA (*p* < 0.001, *χ*^2^ = 12.6); mean dosage of initial antipsychotic treatment (n.s.); route of admin at admission (n.s.); depot vs. oral AP at discharge (*p* < 0.05, *χ*^2^ = 6.3)	
	12. Acosta et al. (2009)	Amador Insight scale, PANSS	Age, sex, marital status, education level, living alone or with someone, length of illness, number of prior hospitalizations, time since last hospitalization (all n.s.)	PANSS conceptual disorganization (OR = 1.74, CI 0.96–3.17, *p* = 0.068); present and past substance use or abuse (n.s.); poor insight (OR = 1.22, 95% CI 1.01–1.48, *p* = 0.040)	Medication class and dosage (n.s.)	
	13. Yang et al. (2012)	CDSS; CGI; PANSS; LUNSERS; DAI; SWN; Revised Insight Scale for Psychosis; WIS	All n.s.	CDSS (n.s.); CGI-S at baseline (*r* = −0.301, *p* < 0.050); CGI-S at 4 w (*r* = −0.403, *p* < 0.010); CGI-S at 8 w (*r* = −0.426, *p* < 0.010); PANSS score excitement [mean (SD), adherent 1.23 (0.43) vs. non-adherent 1.63 (0.83), p = 0.032], poor impulse control [mean (SD), adherent 1.23 (0.43) vs. non-adherent 1.58 (0.77), *p* = 0.049], and preoccupation [mean (SD), adherent 1.27 (0.58) vs. non-adherent 1.74(0.93), *p* = 0.035]; neurocognitive functions and insight (n.s.); attitudes toward medication (*r* = 0.49, *p* < 0.010)	Side effects (n.s.); polypharmacy (*r* = 0.358, *p* < 0.050);	Lower perceived support from significant other (only significant in parts of analysis; mean (SD), adherent 3.49 (1.54) vs. non-adherent 4.59 (1.62), *p* = 0.017);
CT—cross-sectional	14. Klingberg et al. (2008)	PANSS, GAF, SCL-GSI; UKU; EPS; AIMS	All n.s.	PANSS, GAF, SCL-GSI, global functioning and neurocognitive function (all n.s); lack of insight (OR = 0.41, 95% CI 0.183–0.915, *p* = 0.030); positive attitude toward medication (*r* = 0.382; *p* < 0.001)	Medication class and dosage (n.s.)	Frequency social contact, patient has a close friend, contact to relatives >10 h per week, influence family criticism, resignation and overprotection (all n.s.)
	15. Mutsatsa et al. (2003)	LUNSERS; ROMI; SAI; SWN; PANSS		Negative symptoms (*t* = −1.98, *p* = 0.050); disorganization (*t* = −2.01, *p* = 0.050); alcohol or non-alcohol substance misuse (n.s.); poor insight (*t* = 5.71, *p* < 0.001); negative attitudes toward medication (*t* = 3.01, *p* = 0.003)	Akathisia, parkinsonism, non-neurological side effects and subjective well-being (all n.s.)	
	16. Bayle et al. (2015)	CGI; PANSS	Age <40 years (OR = 1.566, 95% CI 1.313–1.869, *p* < 0.001); diagnosis of schizophrenia (*p* = 0.008, *χ*^2^ test, adherent 43.7% vs. non-adherent 56.3%); sex, marital status, and living arrangements or occupation (all n.s.)	CGI-S ≥4 (OR = 1.986, 95% CI 1.518–2.598, *p* < 0.0001); lower insight (PANSS-G12) (OR = 1.459, 95% CI 1.225–1.738, *p* < 0.001)		
	17. Molteni et al. (2014)	SE using DAI-30		Positive subjective experience with medication (DAI-30) (OR = 1.10, *p* = 0.002)		
	18. Day et al. (2005)	PANSS; LUNSERS; attitude (DAI, Van Putten, Morisky); BIS; relationship with staff; admission experience		Attitude toward medication (*r* = 0.26, *p* = 0.001)		PEESSS (*r* = 0.73, *p* < 0.001); PEESSC (*r* = 0.79, *p* < 0.001); PEESSI (*r* = 0.16, *p* < 0.001)
	19. Meier et al. (2010)	Illness history (CSSRI); BPRS; GAF; MHS; LUNSERS; DAI	Age, marital status, and living arrangements or occupation gender (all n.s.)	Symptom scales (all n.s.); positive attitude to psychotropic medication (for clinician-rated adherence; *T* = 3.46; *p* < 0.001)	Side effects (n.s.); medication class (n.s.)	
	20. Borras et al. (2007)	PANSS; CGI; “Multidimensional Measurement of Religiousness/Spirituality for Use in Health Research,” the “Religious Coping Index,” and a questionnaire on spiritual and religious adjustment to life events		PANSS positive symptoms (OR = 0.91, 95% CI 0.84–0.98, *p* < 0.001); substance abuse (OR = 4.0, 95% CI 1.5–10.6, *p* < 0.001)		Positively influenced by spiritual beliefs (31%); negatively influenced by spiritual beliefs (26%);
	21. Aldebot and de Mamani (2009)	BPRS; denial coping from COPE inventory	Gender, ethnicity, years of education (n.s.)	BPRS (n.s.); acceptance (n.s.); denial coping (*t* = −2.83, *p* = 0.008)		
	22. McCabe et al. (2012)	PANSS; therapeutic alliance (Helping Alliance Scale)		PANSS total score (OR = 0.984, 95% CI 0.971–0.996, *p* = 0.014)		Therapeutic relationship (clinician-rated OR = 1.51, 95% CI 1.01–2.25, *p* = 0.042; patient-rated OR = 1.35, 95% CI 0.95–1.90, n.s.)
	23. (Jonsdottir et al., 2013)	PANSS; IDS; YMRS; BIS; UKU; NART; WIS; WASI; Bergen n-back test; DKEFS; WMS; CVLT	Age, gender, marital status, education (all n.s.); BMI full adherence > partial adherence (*p* = 0.012)	PANSS n.s.; IDS n.s.; YMRS n.s.; insight: BIS no adherence < full adherence (*p* = 0.013); neurocognition: WAIS n.s.; NART n.s.; WASI no adherence > full adherence *p* < 0.05; WMS and CVLT no adherence > full and partial adherence *p* < 0.05;executive functioning: DKEFS no adherence > full adherence *p* < 0.05; lifetime diagnosis of addiction or abuse of illicit drugs and alcohol partial adherence > full adherence (*p* = 0.000)	SE: UKU poor adherence significant for diarrhea, nausea, and orthostatism (p-value NR)	

**Table 3:** Overview of risk-factors and predictors of antipsychotic medication adherence and non-adherence.

**Predictors**		**Sociodemographics**	**Clinical factors**	**Substance use and abuse**	**Insight and attitude**	**Treatment related**	**Family involvement and therapeutic relations**
Commonly involved	In adherence				Illness insight (8; 10)		Family involvement and support (14; 10; 6)
	In non-adherence	Younger age (7; 9; 16)			Lack of insight (7; 15; 14; 12; 9; 16); positive attitude (18; 19; 13; 9; 17; 14)		
Possibly involved	In adherence				Change toward more positive attitudes (8)		Positive attitude of family members toward medication (10)
	In non-adherence	Age at illness onset (7; 14)	Paranoia (11); hostility (1); excitement (13); poor impulse control and preoccupation (13); poor premorbid functioning (7)	Cannabis (7); comorbid substance dependence syndrome (11; 9)	Negative attitude (15; 9; 13)		Lack of family involvement (7)
Insufficient evidence	In adherence		Subjective well-being (15), neurocognitive functioning (13;14)	Absence of cannabis use (20)	Positive change in insight (8), lower score on “lack of insight” (14)		Therapeutic environment (10; 22); admission experience with regard to psychiatric care (18)
	In non-adherence	Ethnic minorities (6;21)	Positive symptoms (7; 20; 14), negative symptoms (15; 11; 14), poor QoL and high relapse rate (7), disorganization syndromes (15; 12), illness severity (2; 14; 21; 19; 22; 13;16), depressive symptoms (1; 13); denial coping (21), comorbid harm or dependence syndrome (9); mode of admission (10; 11); number of previous admissions (11; 12); global functioning (9; 14;19)	Substance use (15; 20; 1; 6); alcohol (7; 15)	Lower positive attitude (15; 9)	Administration route (11; 14; 3); EPS (15; 11; 6), weight change (1), non-neurological SE (15), adverse events (1; 19; 10; 13)	Living with family with high EE (2)
Low evidence	In adherence	Gender (11; 2; 14; 12; 16); occupation (14; 16), marital status (12; 16); level of education (11; 14; 12); duration of illness (11; 12; 10); illness characteristics (1)	Treatment efficacy (1)			Medication class (11; 4; 14; 19; 10; 5); mean AP dosage (11; 14; 12; 13)	
	In non-adherence		Problem-solving ability (14)	History of substance abuse (12; 1)			

##### Sociodemographic Risk Factors

Evidence from 13 studies assessing the relation between sociodemographic risk factors or predictors and adherence are summarized below.

One randomized controlled study (RCT) with 599 patients with schizophrenia and schizoaffective disorders (Lindenmayer et al., 2009), two cross-sectional studies (Meier et al., 2010; Jonsdottir et al., 2013), and two longitudinal cohorts (Acosta et al., 2009; Yang et al., 2012) investigated baseline demographics as potential risk factors but found none to be good predictors of non-adherence. Results were mixed concerning age as a predictor. Both younger age and, to a lesser extent, also younger age at illness onset have been identified as a strong predictor of non-adherence, although other studies have failed to replicate this finding. Findings were mixed regarding adherence rates in ethnic minorities compared to Caucasian patients (Aldebot and de Mamani, 2009; Winton-Brown et al., 2017). Furthermore, adherence behavior is not related to patients' marital status (Acosta et al., 2009; Higashi et al., 2013; Jonsdottir et al., 2013; Bayle et al., 2015; Sendt et al., 2015), gender (Janssen et al., 2006; Morken et al., 2007; Klingberg et al., 2008; Acosta et al., 2009; Aldebot and de Mamani, 2009; Higashi et al., 2013; Jonsdottir et al., 2013; Bayle et al., 2015; Sendt et al., 2015), occupation (Klingberg et al., 2008; Higashi et al., 2013; Bayle et al., 2015; Sendt et al., 2015), and level of education (Klingberg et al., 2008; Acosta et al., 2009; Aldebot and de Mamani, 2009; Higashi et al., 2013; Jonsdottir et al., 2013; Sendt et al., 2015), with the exception of one longitudinal study that found a small association with non-adherence (OR, 0.59; 95% CI, 0.41–0.86, *p* < 0.01) (Janssen et al., 2006).

##### Clinical Risk Factors

Twenty studies investigated the relation between symptom severity and antipsychotic adherence behavior. While a significant association between increasing severity of illness and decreasing antipsychotic adherence was reported in four individual studies (Morken et al., 2007; McCabe et al., 2012; Yang et al., 2012; Bayle et al., 2015), no association of symptom severity was reported in three others (Klingberg et al., 2008; Aldebot and de Mamani, 2009; Meier et al., 2010; Jonsdottir et al., 2013). However, generalization of the results is complicated by the fact that symptoms were assessed using different scales, i.e., Positive and Negative Syndrome Scale (PANSS), Clinical Global impression (CGI) scale, and Brief Psychiatric Rating Scale (BPRS).

Positive symptoms have been linked to non-adherence in a longitudinal cohort of patients with first-episode psychosis (FEP) (Coldham et al., 2002) and in stable patients (Borras et al., 2007). However, no significant association with adherence was observed in another cohort of stable patients (Klingberg et al., 2008). In addition, high intensity of excitement (Yang et al., 2012), hostility (Lindenmayer et al., 2009), and a high PANSS paranoid subscore (Janssen et al., 2006) were also identified as risk factors of non-adherence, while higher scores on disorganization syndromes (Mutsatsa et al., 2003; Acosta et al., 2009) were weak predictors of non-adherence. Evidence for other factors such as a higher negative subscore on the PANSS (Mutsatsa et al., 2003; Janssen et al., 2006; Klingberg et al., 2008) was weak, while poor impulse control and preoccupation have been associated with non-adherence (Yang et al., 2012). A significant association has also been found between both depressive symptoms as measured by the MADRS (total score, *p* = 0.01; reported sadness, *p* = 0.04; pessimistic thoughts, *p* = 0.01) and the PANSS (depressive factor HR = 1.2; 95% CI, 1.06–1.35; *p* = 0.003), in an RCT (Lindenmayer et al., 2009). In contrast, no association for depressive symptoms as measured by the CDSS and IDS was found in a longitudinal (Yang et al., 2012) and cross-sectional cohort (Jonsdottir et al., 2013), respectively. Although the design and included sample size of the prospective cohort generates limited evidence, the CDSS can differentiate depressive symptoms more accurately from other symptoms (Lako et al., 2012) compared to the MADRS. No association was found for manic symptoms as measured by the YMRS and adherence (Jonsdottir et al., 2013).

Furthermore, illness characteristics (Lindenmayer et al., 2009), including specific diagnosis (Janssen et al., 2006; Klingberg et al., 2008; Baloush-Kleinman et al., 2011; Bayle et al., 2015), and duration of illness (Janssen et al., 2006; Acosta et al., 2009; Baloush-Kleinman et al., 2011; Sendt et al., 2015) were poor predictors for adherence behavior. Other factors, such as denial coping (Aldebot and de Mamani, 2009), comorbid harm or dependence syndrome (Quach et al., 2009), poorer impulse control, poorer quality of life, and higher relapse rate (Coldham et al., 2002) were also weakly associated with non-adherence. Surprisingly, higher subjective well-being (Mutsatsa et al., 2003) and treatment efficacy (PANSS total score, *p* = 0.38) (Lindenmayer et al., 2009) do not predict adherence. With the exception of poor premorbid functioning in FEP (Coldham et al., 2002), general functioning, including current score on the Global Assessment of Functioning (GAF) scale and self-rated problem-solving ability (Klingberg et al., 2008), was not predictive of non-adherence. Additionally, neurocognitive function domains, including IQ, as assessed by the Wechsler Intelligence Scale, executive functioning (verbal fluency and trail making test) (Yang et al., 2012), working memory, and attention (Klingberg et al., 2008) did not predict adherence, and one study even found an inverse relationship between neurocognitive functioning and adherence (Jonsdottir et al., 2013).

Findings regarding previous psychiatric hospitalizations were mixed. Mode of admission (*n* = 112) (Baloush-Kleinman et al., 2011), number of prior admissions, and time since last hospitalization (*n* = 74) (Acosta et al., 2009) were reported to be not significant as predictors in two longitudinal trials. Another study with a larger sample size (*n* = 670) found that (Janssen et al., 2006) while first or multiple episode admission were not different in predicting non-adherence, the number of previous admissions and involuntary admission were significantly predictive of non-adherence.

##### Substance Use

Although a dual diagnosis of substance dependence syndrome as comorbidity to psychosis has been associated with poor adherence (Janssen et al., 2006; Quach et al., 2009; Jonsdottir et al., 2013), evidence is lacking for both alcohol abuse and illicit substance abuse as reliable individual predictors of medication non-adherence.

In FEP, a longitudinal cohort demonstrated significantly higher levels of alcohol use in the non-adherent group (Coldham et al., 2002). No significant association between non-adherence and alcohol was found elsewhere (Mutsatsa et al., 2003).

Misuse of illicit substances has been significantly associated with poor adherence in two studies (Winton-Brown et al., 2017) but refuted elsewhere (Mutsatsa et al., 2003; Acosta et al., 2009; Lindenmayer et al., 2009). Unsurprisingly, among different substances, cannabis—the most used illicit drug among patients with psychosis—was the strongest predictor of non-adherence to antipsychotic medication (Coldham et al., 2002), and absence of cannabis use was predictive of adherence (Borras et al., 2007).

##### Illness Insight and Medication Attitudes

Some of the most consistent results were found for the relationship between low illness insight and adherence. It is proposed that because patients with psychosis lack insight into their disease, this affects adherence to their medication regimes. Indeed, lack of insight, including unawareness of the effect of medication and negative medication beliefs, were significantly associated with medication non-adherence in all but one study (Yang et al., 2012). This finding was consistent over the different illness stages: in FEP (Coldham et al., 2002; Mutsatsa et al., 2003; Quach et al., 2009), in patients with a recent acute psychotic episode (Bayle et al., 2015), and in clinically stable patients (Klingberg et al., 2008; Acosta et al., 2009; Mohamed et al., 2009). Positive change in insight scores also predicted adherence in clinically stable patients (Mohamed et al., 2009). In the same line, illness insight (Mohamed et al., 2009; Baloush-Kleinman et al., 2011), including better awareness of the need for treatment and social consequences of illness (Baloush-Kleinman et al., 2011), is a consistent predictor of good adherence.

Unsurprisingly, an overall positive attitude toward antipsychotic medication is highly associated with adherence (Day et al., 2005; Quach et al., 2009; Meier et al., 2010; Yang et al., 2012), a finding that has been replicated in adolescents with psychosis (Molteni et al., 2014), and in clinically stable patients with schizophrenia and schizoaffective disorders (Klingberg et al., 2008). In addition, a change toward more positive attitudes (Mohamed et al., 2009) was correlated with greater medication adherence. Inconsistent findings were reported for lack of positive attitude and medication adherence in FEP. Although lower positive attitude has been found to be unrelated to adherence in one study (Mutsatsa et al., 2003), lack of positive attitude was identified as a predictor of antipsychotics non-adherence in another (Quach et al., 2009). In this line, negative attitude toward antipsychotic medication may be a relevant predictor of poor adherence to antipsychotic medication (Yang et al., 2012), particularly among patients with FEP (Mutsatsa et al., 2003; Quach et al., 2009). In addition, a study reported on the direct impact of spiritual beliefs adherence and found that 26% were negatively and 31% positively influenced by their spiritual beliefs (Borras et al., 2007).

#### Treatment-Related Factors

Factors related to antipsychotic treatment, such as type, dosage, and route of medication administration are difficult to evaluate reliably outside of RCTs due to the confounding effect of clinical characteristics occurring in naturalistic studies.

One prospective cohort of 670 subjects (Janssen et al., 2006) found that patients using second-generation antipsychotics (SGA) monopharmacy had better adherence at discharge than patients using first-generation antipsychotics (FGA) either as monotherapy or in combination. In addition, those on FGA monotherapy who switched to an SGA (55 %) also had a significantly higher good adherence rate at discharge than those who had continued to take FGA medication, which, according to the authors, may be explained by the prescribers' preference for SGAs in patients with better adherence. Interestingly, the finding that antipsychotic medication class was associated with adherence rates has not been replicated in an open RCT of haloperidol vs. SGAs in patients with FEP (Kahn et al., 2008) nor in open-label (SGA vs. FGA) (Guo et al., 2011), cross-sectional (Klingberg et al., 2008; Meier et al., 2010), and longitudinal setting (Baloush-Kleinman et al., 2011) with stable schizophrenia patients.

On the same note, giving patients control over the choice of route of antipsychotic medication administration did not lead to better adherence in an RCT of intramuscular vs. oral antipsychotics in FEP (Weiden et al., 2012). In addition, administration route of medication in stable subjects did not significantly impact adherence (Janssen et al., 2006). If anything, patients prescribed with antipsychotic depot formulations at discharge even had a significantly higher non-adherence rate (34.7% of *n* = 149) compared to those on oral medication (48.4% of *n* = 521; *p* < 0.05). Yet, in such a naturalistic setting, obviously considerable selection bias would exist with clinicians being more likely to prescribe depot formulations in patients considered *a priori* to be at risk for non-adherence. Finally, the mean dosage of antipsychotic treatment did not influence adherence behavior in the reviewed studies. One small-sized cohort did report a correlation of non-adherence with polypharmacy of antipsychotic drugs (*r* = 0.358, *p* < 0.05) (Yang et al., 2012).

Although low tolerability of antipsychotic medication is often viewed as an important reason for non-adherence, medication side effects do not seem to carry strong predictive effects. Two individual cohorts found no association between antipsychotic-induced side effects and medication adherence behavior (Meier et al., 2010; Yang et al., 2012), while one identified side effects as an impediment to adherence. Weight change has been demonstrated to be a poor predictor of non-adherence (Lindenmayer et al., 2009), and extrapyramidal side effects (EPS), such as akathisia and parkinsonism, significantly predicted medication non-adherence in some but not all studies (Mutsatsa et al., 2003; Winton-Brown et al., 2017). Unexpectedly, adherence did not differ between patients with EPS compared to those without (47.8% adherent vs. 41.3% non-adherent) in a study of inpatients of different illness stages (Janssen et al., 2006). Moreover, no non-neurological side effects were reported to be significant (Mutsatsa et al., 2003). Overall tolerability, measured by the maximum severity of adverse effects, was a poor predictor of non-adherence (Lindenmayer et al., 2009).

#### Family Involvement and Therapeutic Relations

The relative contribution of social and family involvement and therapeutic relations to medication adherence is suggested to be highly relevant. Indeed, higher level of family and career involvement and support (Baloush-Kleinman et al., 2011; Winton-Brown et al., 2017) and positive attitudes of family members toward medication (Baloush-Kleinman et al., 2011) are good predictors of medication adherence. One study failed to find an association between medication adherence and “expressed emotions” (i.e., degree of criticism, resignation, and overprotection expressed by relatives) (Klingberg et al., 2008). This may be explained by the inclusion of patients of different illness stages, as one systematic review emphasized that social support and family involvement are particularly beneficial for adherence in younger study populations (Sendt et al., 2015). In addition, another study suggested living with family with high expressed emotions was associated with higher adherence rates (Morken et al., 2007). Moreover, lack of family involvement and social support was also found to be predictive of poor adherence to antipsychotic treatment (Coldham et al., 2002; Yang et al., 2012). One longitudinal study reported that patients who were not upbrought by both parents or had no key relative that came to entry interview were at greater risk of medication non-adherence (Quach et al., 2009). The quality of the therapeutic relationship, as rated by both patients and clinicians (Baloush-Kleinman et al., 2011; McCabe et al., 2012), can indirectly influence adherence by mediating better attitudes to medication (Sendt et al., 2015) or to the psychiatric care in general (Day et al., 2005).

### Interventions to Improve Antipsychotic Medication Adherence

We identified 17 distinct studies involving individuals with psychotic spectrum disorder undergoing an intervention to improve antipsychotic medication adherence. Four main intervention groups were identified: behavioral interventions, family interventions, LAI + interventions, and technology interventions (see Table 4). Objective adherence measures included pill counts, prescription refill rates, or blood plasma concentration levels. Subjective clinician-rated or self-reported measures quantifying medication adherence were also eligible (e.g., Medication Adherence Questionnaire).

**Table 4:** Evidence table on interventions to improve medication adherence.

**Intervention type**	**Study**	**Study type**	**Sample characteristics**	**Cases**	**Duration of study**	**Adherence measure**	**Effect on adherence**	**Effect other outcome measures**	**RoB**
Behavioral—adherence therapy	Anderson et al. (2010)	RCT, SB	Mixed (%FEP NR); stable; SZ + SZA	12 intervention vs. 14 TAU	8 w	PETiT	*t* = 1.20, n.s.		Low
	Chien et al. (2015)	RCT, SB	Mixed (%FEP NR); stable; PSD	57 intervention vs. 57 TAU	4 m; FU at 6 m	ARS	*F* = 7.45, *p* = 0.007; ES = 0.72	PANSS score (*F* = 7.32, *p* = 0.008); positive symptoms score (*F* = 7.28, *p* = 0.008); negative symptoms score (*F* = 7.81, *p* = 0.006); ES = 0.70–0.75; number of rehospitalizations (*F* = 5.01, *p* = 0.030), ES = 0.48; insight into illness and/or treatment (*F* = 6.58, *p* = 0.021), ES = 0.51; functioning (*F* = 6.89, *p* = 0.014), ES = 0.68	Low
	Chien et al. (2016)	RCT, SB	Mixed (%FEP NR); stable; PSD	67 intervention vs. 67 TAU	12 w; 18 m FU (2w, 6m, 18m)	ARS	Non-adherent: 85 vs. 90% (*F* = 9.10, *p* = 0.005), effect size = 0.30	Insight (*F* = 10.98, *p* = 0.001), ES = 0.40; functioning (*F* = 8.90, *p* = 0.005), ES = 0.29; symptom severity (PANSS) (*F* = 10.10, *p* = 0.003), ES = 0.32, hospital rate duration (*F* = 8.80, *p* = 0.005), ES = 0.28; hospital rate frequency (*F* = 3.47, *p* = 0.092)	Low
	Gray et al. (2006)	RCT, SB	Chronic; state NR; SZ	204 intervention vs. 205 HE (control)	52 w (8 weekly sessions within first 5 m)	MAQ, SAI-C	MAQ: n.s.; SAI-C: -n.s.	n.s. QoL and BPRS	Low
	Schulz et al. (2013)	RCT, SB	Mixed (%FEP NR); acute; SZ	80 intervention vs. 57 TAU	12 w	CDR, MARS	CDR: *F* = 2.29, n.s.; MARS: difference 0	PANSS (*F* = 6.19, *p* < 0.05); beliefs about treatment (DAI) n.s.; GAF n.s.	Low
Behavioral—CBT	Bechdolf et al. (2010)	RCT, SB	Mixed (% FEP NR); acute; PSD	16 CBT vs. 27 PE	8 w, results FU at 24 m	4-point rating scale	*F* = 1.31, *p* = 0.26	Rehospitalization rate 37.5% vs. 59.3%, (*χ*^2^ = 2.50, n.s.); symptom severity n.s.	Low
Behavioral—cognitive adaptation training	Velligan et al. (2008)	RCT, SB	Chronic; stable; SZ + SZA	34 CAT vs. 32 PharmCAT vs. 29 TAU	9 + 6 m FU (3 and 6 m)	Unannounced in-home pill counts; prescription refill rates	Pill count adherence: CAT vs. TAU ES = 1.09; Pharm-CAT vs. TAU ES = 1.05; prescription refill rates: main effect of group (*F* = 3.93, *p* < 0.020), CAT vs. TAU (*F* = −2.85, *p* < 0.006), Pharm-CAT vs. TAU n.s.; CAT vs. TAU ES = 0.51 and Pharm-CAT vs. TAU ES = 0.33	Symptom severity n.s.; relapse rate CAT vs. TAU (*χ*^2^ = 8.29, *p* < 0.004); Pharm-CAT vs. TAU (*χ*^2^ = 8.20, *p* < 0.005); relapse in 15 m >65% CAT and Pharm-CAT vs. 19% TAU; functional outcome CAT vs. TAU 6 m treatment ES = 1.47 and 6 m FU ES = 0.50, Pharm-CAT vs. TAU at 3 m ES = 0.42, at 6 m treatment ES = 0.44, at 6 m FU ES = 0.22	Low
	Velligan et al. (2013)	RCT, SB	Chronic; stable; SZ + SZA	46 MeM vs. 46 PharmCAT vs. 45 TAU	9 m	Electronic monitor, pill counts	e-monitoring: treatment group effect *F* = 47.29, *p* < 0.0001; effects for time *F* = 0.06, n.s.; time × group effect *F* = 0.44, n.s.; PharmCAT vs. TAU ES = 1.03 and MeM vs. TAU ES = 0.98. Pill counts: significant main effect of group *F* = 7.83, *p* < 0.0001 and n.s. effects of time *F* = <1, n.s.; time × group interaction *F* = 2.34, *p* = 0.06; adherence rate PharmCAT 91% vs. MeM 86%, *t* = 2.05, *p* = 0.04; PHARMCAT 91% vs. TAU 80%, *t* = 3.95, *p* = 0.0001; MeM 86% vs. TAU 80%, *t* = 1.82, n.s.	Symptom severity and functioning (all n.s.)	Low
Family therapy	Kopelowicz et al. (2012)	RCT, SB	Mixed (%FEP NR); stable; SZ + SZA	64 MFG-adherence vs. 53 MFG-standard vs. 57 TAU	12 m (FU at 18 m and 24 m)	Treatment Compliance Interview	Group effect (*F* = 6.41, *p* = 0.003); Time effect (*F* = 3.5, *p* = 0.009); Group × time effect n.s.	Group differences in time to first hospitalization (*χ*^2^ = 13.3, *p* = 0.001); at FU MFG-A vs. MFG-S (*χ*^2^ = 6.3, *p* = 0.01) and MFG-A vs. TAU (*χ*^2^ = 8.7, *p* = 0.003); hospitalization rate: MFG-A (39%) vs. MFG-S (66%) (*χ*^2^ = 8.2, *p* = 0.004), MFG-A vs TAU (70.2%) (χ^2^ = 11.3, *p* < 0.001); MFG-S vs. TAU (*χ*^2^ = 0.2, n.s.)	Low
	Valencia et al. (2010)	RCT, SB	Mixed (%FEP NR); stable; SZ	47 intervention vs. 36 TAU	12 m	Prescription renewals, patient's and key relative's monthly report to the treating psychiatrist	Medication adherence 91.5 vs. 77.8% (*p* < 0.050); visit adherence 82.5 vs. 70% (*p* < 0.050)	Global functioning ES = 1.30 vs. TAU ES 0.30 (effect for time, group and time × group all *p* < 0.010); relapse rate 12.8 vs. 33.3%, *p* < 0.05; rehospitalization 2.1 vs. 14%, *p* < 0.050	Low
LAI	Noordraven et al. (2017)	Open label RCT	Chronic; stable; PSD	84 intervention vs. 85 TAU	12 m (+6 m FU)	MPR, longest uninterrupted period during which depot medication was received, time to first discontinuation of depot medication, total number of days without depot medication, and time between prescription date and the date the depot was actually received	MPR 14.9% (95% CI 8.9–20.9), *p* < 0.0001; good adherence (MPR ≥80 %) = 33.1% (95% CI 20.2–45.4), p = 0.031; 6 m FU MPR 6.5% (95% CI 2.0–10.9), *p* = 0.047; 6 m FU good adherence: 22.1% (95% CI 4.2–39.8%), *p* = 0.010	Attitudes, clinical symptoms, psychosocial functioning, substance use, QoL, side effects (all n.s.)	Moderate
	Lee et al. (2010)	CT—prospective, controlled, unrandomized	Mixed (% FEP NR); stable; SZ + SZA	21 intervention vs. 25 TAU	12 m (+FU at 2 y)	Visits for injection/planned visits for injection; treatment discontinuation; injection discontinuation	1 y FU intervention: 94.6%, TAU: 75.9%, (*t* = 3.5, *p* < 0.010); 2 y FU intervention: 92.1%, TAU: 74.2%, (*t* = 2.7, *p* < 0.010); treatment discontinuation: intervention 14% vs. TAU 28% (*χ*^2^ = 6.0, *p* = 0.010); injection discontinuation: intervention 23% vs. TAU 68% (*χ*^2^ = 13.0, *p* < 0.010)	1 y relapse rate intervention vs. TAU *p* < 0.010; 2 y relapse rate intervention vs. TAU *χ*^2^ = 4.2, *p* = 0.040; symptom severity n.s.; side effects n.s	Moderate
	Sajatovic et al. (2013)	CT—prospective, uncontrolled trial	Mixed (% FEP NR); state NR; SZ + SZA	30	6 m	TRQ, MAQ, injection frequency	TRQ (incl. oral medication, mean) −38.9 (95% CI, −75.7–−2.0), *p* = 0.028; MAQ, mean (SD): 1.4 (1.6), *p* = 0.001; injection frequency, mean (SD): only at week 13: 83 (35), and week 25: 76 (35)	Improvements in psychiatric symptoms (*p* < 0.001; BPRS (*t* = 2.51, *p* = 0.029), PANSS (*p* = 0.005), CGI (*p* < 0.001), and functioning (*p* < 0.001), akathisia (40%); BMI and total cholesterol n.s.; changes in hospitalizations n.s.	Low
Technology	Frangou et al. (2005)	RCT, open	Chronic; stable; SZ	36 pill counting vs. 36 @HOME vs. 36 TAU	8 w	MAQ-based questionnaire; pill counting; e-monitoring (incl. electronic dispenser)	TAU, mean (SD; range)%: 77.3 (22.1; 18–95)%; pill counting, mean (SD; range)% = 78.5% (14; 50–95); e-monitoring, mean (SD; range)%: mean of 92.3% (4.8; 82–100); effect of group (*F* = 8.9, *p* = 0.0001); TAU vs. pill counting (n.s.); e-monitoring group vs. TAU (*p* = 0.001); e-monitoring vs. pill counting group (*p* = 0.007)	Group differences in the PANSS total score (*F* = 5.7, *p* = 0.004); control vs. pill-counting group (*p* = 0.008) and e-monitoring (*p* = 0.04); pill-counting vs. e-monitoring (*p* = 0.8); end-point medical (*p* = 0.01) and emergency (*p* = 0.0001) visits in the @HOME patient, group difference (*F* = 3.6, *p* = 0.002)	Moderate
	Montes et al. (2012)	RCT; open	Chronic; stable; SZ	100 intervention vs. 154 TAU	6 m (3 and 6 m)	MAQ	MAQ [mean (95% CI)] 3 m: mean total score change intervention-−1.0 (−1.02–−0.98) vs. TAU −0.7 (−0.72–−0.68) *p* = 0.02; 6 m: mean total score change intervention-−1.1 (−1.12–−1.08) vs. TAU 0.8 (0.81, 0.78), *p* = 0.04	Symptom improvement [mean (95% CI)] 3 m: improvement in negative [intervention 3.3 (3.10–3.50) vs. TAU 3.5 (3.36–3.64), *p* = 0.020], cognitive [intervention 3.3 (3.12–3.48) vs. TAU 3.6 (3.46–3.74), *p* = 0.010] and global [intervention 3.2 (3.02–3.38) vs. TAU 3.5 (3.36–3.64), *p* = 0.012) symptoms; 6 m negative (n.s.), cognitive (n.s.) and global (n.s.) symptoms; attitude [mean (95% CI)] 3 m: intervention 2.0 (1.94, 2.06), vs. TAU 0.4 (0.35, 0.45), *p* = 0.0003; 6 m: intervention 2.3 (2.24, 2.36), vs. TAU 0.9 (0.85, 0.95), *p* = 0.002; insight n.s.; QoL intervention 6.6 (6.38–6.82) vs. TAU 3.1 (2.91–3.29), *p* < 0.03; 6 m: n.s.	Moderate
	Velligan et al. (2013)	RCT, SB	Chronic; stable; SZ + SZA	46 MeM vs. 46 PharmCAT vs. 45 TAU	9 m	Electronic monitor, pill counts	e-monitoring: treatment group effect *F* = 47.29, *p* < 0.0001; effects for time *F* = 0.06, n.s.; time × group effect *F* = 0.44, n.s.; PharmCAT vs. TAU ES = 1.03 and MeM vs. TAU ES = 0.98. Pill counts: significant main effect of group *F* = 7.83, *p* < 0.0001 and n.s. effects of time *F* = <1, n.s.; time × group interaction *F* = 2.34, *p* = 0.06; adherence rate PharmCAT 91% vs. MeM 86%, *t* = 2.05, *p* = 0.04; PHARMCAT 91% vs. TAU 80%, *t* = 3.95, *p* = 0.0001; MeM 86% vs. TAU 80%, *t* = 1.82, n.s.	All n.s. (*p* > 0.090; symptom severity and functioning)	Low
	Moncrieff et al. (2016)	RCT, open	Mixed (% FEP NR); state NR; PSD	31 intervention vs. 29 TAU	3m (FU 2–3 w; 2–3 m)	MAQ	OR = −0.44, 95% CI, −0.76–−0.11	Positive attitudes to antipsychotic medication (DAI, 1.65; 95% CI, −0.09–3.40); PANSS, side effects and dosage (all n.s.)	Moderate
	Beebe et al. (2017)	RCT, SB	Mixed (% FEP NR); stable; SZ + SZA	53 intervention vs. 52 TAU	6 m	Pill counts; serum medication levels	Pill counts adherence: 66% vs. 50%, (*χ*^2^, n.s.); serum AP levels within therapeutic range: 54.7% vs. 32.7% (*χ*^2^ = 5.2, *p* = 0.023)		Low

#### Behavioral Interventions

##### Adherence Therapy

Adherence therapy (AT) is a 12-session patient-centered therapy that mainly involves a combination of techniques derived from motivational interviewing, cognitive behavioral therapy, and psychoeducation to promote treatment adherence (Kemp et al., 1996). All five included individual studies here employed the modified, brief (six to eight sessions) course designed by Gray et al. (2006). Mixed findings were demonstrated concerning the efficacy of AT in terms of improving adherence. Antipsychotic medication adherence was measured with different tools in all five studies, with four using only subjective measures (Gray et al., 2006; Anderson et al., 2010; Chien et al., 2015, 2016) and one combining subjective and objective tools (Schulz et al., 2013).

No significant differences in adherence behavior between the intervention and control group was found in three single-blind RCTs (Gray et al., 2006; Anderson et al., 2010; Schulz et al., 2013), irrespective of outcome measure used. AT was not found to be more effective than health education in improving participant's adherence to medication and quality of life (measured by different self-rating scales) after the intervention or at 1-year follow-up (total *n* = 409) (Gray et al., 2006). AT did also not significantly affect patients' adherence and treatment attitudes in a study using both subjective and objective (serum concentrations of antipsychotic medication) measures for adherence. Yet, despite the lack of improvement in adherence in this study, the symptom severity scores improved significantly more in the AT group compared to treatment-as-usual (TAU) (Schulz et al., 2013). We cannot exclude the possibility that selection bias (of patients with positive medication attitudes), ceiling effects (high mean baseline CDR levels), and a lack of power may have obscured any effect of the intervention in Anderson et al. (2010) and Schulz et al. (2013).

Only two out of five studies, both of them conducted by the same research group, found AT to be effective in improving medication adherence at small-to-large effect sizes (effect size, 0.72 and 0.30) (Chien et al., 2015, 2016). Both of these studies [*n* = 114 (Chien et al., 2015) and *n* = 134 (Chien et al., 2016)] featured a slightly modified treatment with a larger proportion of motivational interviewing techniques. Along with a significantly greater improvement over time in medication adherence of the AT group, there was also a significantly greater improvement of symptom severity, illness insight, global functioning, and rate of hospitalization at 6-month follow-up. Importantly, the study that found the larger effect size only included previously non-adherent patients and had a very low (7%) refusal rate as well as a high family support; which may all have inflated the results.

##### CBT

Only one RCT has studied (Bechdolf et al., 2005) group cognitive behavioral therapy (CBT) vs. psychoeducation (PE) group training for medication adherence. The group CBT treatment consisted of coping strategy enhancement, problem solving, and relapse prevention in patients with psychosis. The intervention was focused on the treatment of symptoms, relapse prevention and associated problems, and enhancing medication adherence and included 16 sessions in 8 weeks. The eight PE training sessions were covered in the same time window.

Adherence was measured posttreatment and at the 24-month follow-up, using a 4-point rating scale based on multiple sources, including patients, relatives, and clinical staff. Although no significant differences were reported on adherence levels between the two interventions at any assessment point, both interventions led to relevant clinical improvement, in terms of rehospitalization, symptom severity, and medication use, at the end of treatment and at follow-up. Readmission was not significantly related to non-adherence. Baseline medication adherence was high in both groups, with a mean score of 3.9 ± 0.3 and 3.77 ± 0.5 for the CBT and PE group, respectively, possibly leaving no room for further improvement. Moreover, the author reported that the follow-up sample might have been unrepresentative due to the high lost-to-follow-up rate.

##### Cognitive Adaptation Training

Two studies by the same research group investigated cognitive adaptation training (CAT) for medication adherence in schizophrenia. In one study (Velligan et al., 2008), patients were randomized to receive either CAT, Pharm-CAT, which is a subset of techniques from the CAT program, or TAU. CAT is a series of compensatory strategies and environmental supports designed to improve multiple domains of adaptive functioning including medication adherence, grooming, and independent living skills in patients with schizophrenia (Velligan et al., 2008). Pharm-CAT uses environmental supports such as checklists, signs, and electronic cueing devices to improve medication adherence. In contrast to full CAT treatment, only interventions that specifically target adherence are used (Velligan et al., 2008, 2013). Treatment lasted for 9 months, and follow-up lasted to 6 months after end of treatment. Objective adherence measures in the form of unannounced in-home pill counts and prescription refill rates were used. Adherence and functional outcomes were assessed every 3 months. A superior treatment effect with large effect sizes for both CAT (ES = 1.09) and Pharm-CAT (ES = 1.03) over TAU in pill count adherence was established during intervention and at follow-up, and adherence remained close to 80%. In addition, only small-to-moderate effects were found in prescription fill rate (ES CAT = 0.51 and Pharm-CAT = 0.33). Across the treatment groups, no significant differences in symptom severity were demonstrated. However, relapse rates in the CAT and Pharm-CAT groups were significantly lower than in the TAU group, with no significant differences between the active treatment groups. Pharm-CAT was only significantly different than TAU in improving functioning in the first 6 months of treatment. The authors suggested that this slight improvement in functioning in the group receiving Pharm-CAT may be due to better medication adherence in this group as compared to patients receiving standard treatment.

In another study (Velligan et al., 2013), patients were randomized to receive either standard treatment, Pharm-CAT, or a smart pill container known as the Med-eMonitor for 9 months. Here, adherence was obtained via an electronic monitor and by monthly unannounced pill counts conducted in participants' homes. All groups received a monitoring device to assess adherence, but only in the Med-eMonitor group the monitor was set to encourage adherence. More specifically, the Med-eMonitor was capable of cueing patients to take their medication and warning them when they are taking the wrong medication, documenting adverse events complaints, and alerting clinical staff of failure to adhere to medication. Compared to TAU, medication adherence measured with e-monitoring was significantly higher in both active intervention groups (ES Pharm-CAT = 1.03 and MeM = 0.98). No differences between the Pharm-CAT and Med-eMonitor treatment groups were found. In contrast, medication adherence as measured by pill counting was higher in the Pharm-CAT group (91%) compared to the Med-eMonitor (86%, *p* = 0.04) or TAU group (80%, *p* = 0.0001). Although the active interventions significantly improved medication adherence, this did not translate to improved clinical outcomes in terms of symptom severity or global functioning.

#### Family Interventions

According to two single-blind RCTs, add-on family-based interventions seem to result in better medication adherence as compared to TAU alone. In one study, outpatients were randomized to either continue TAU or receive a 12-month psychosocial rehabilitation, including Psychosocial Skills Training (PSST) and family psychoeducation on top of TAU in one study (Valencia et al., 2010). Subjects' relatives who were randomized to PSST participated in 12 psychoeducational, multifamily group sessions in which they received similar information as the patients. This included providing effective support to the person with schizophrenia and coping with the disorder; information on symptoms, medication, side effects, and the importance of treatment (Kopelowicz and Liberman, 2003). Adherence assessment included both subjective measures by patient and key relative's report and objective measures in the form of prescription renewals. Medication and appointment adherence was significantly greater among patients receiving psychosocial rehabilitation than their counterparts in the TAU condition. Moreover, the addition of PSST and family psychoeducation to antipsychotic medication significantly reduced psychiatric symptoms, relapses and rehospitalization rate, and improved global functioning (Valencia et al., 2010).

Similarly, a 12-month multifamily group (MFG) treatment, a behavioral family treatment that combines psychoeducation and skills training, as earlier described by McFarlane (2002), was employed in another RCT (Kopelowicz et al., 2012). Standard MFG therapy (MFG-S) was compared to both TAU and adherence-focused MFG (MFG-A), which focuses on attitudes, subjective norms, and perceived behavioral control. Adherence was evaluated using Treatment Compliance Interview (Weiden et al., 1995), an instrument that provides a quantified rating of the extent to which the patient did take their medication and the amount of medication they may have taken in the past month. Patient's key relatives were also interviewed using the relative version of the instrument. No significant differences in level of adherence were reported at any point between the MFG-S and control group. However, more participants in MFG-A were fully adherent than those in TAU at all assessments during the treatment but not at the 24-month follow-up. Group differences in time to first hospitalization after baseline was significant: rehospitalization was less likely for those in MFG-A than for those receiving MFG-S or standard treatment across the entire follow-up period (Kopelowicz et al., 2012).

#### LAI Combined With a Psychoeducation-Based or Monetary Intervention

Three studies examining interventions for medication adherence in patients prescribed a long-acting injectable (LAI) antipsychotic were included. One study (Sajatovic et al., 2013) assessed long-acting injectable antipsychotics (haloperidol) in combination with a customized adherence enhancement intervention. This intervention includes psychoeducation focused on medication, developing medication routines, and managing adherence in the context of substance abuse (Sajatovic et al., 2013). Adherence was assessed using both subjective tools [Tablets Routine Questionnaire (Scott and Pope, 2002) and Morisky scale (Morisky et al., 1986)] and objective measures (injection frequency). A significant positive change in both adherence to LAI and concomitant oral antipsychotics was illustrated through the uncontrolled 25-week intervention, as well as symptom severity and social functioning. No significant changes in hospitalizations were reported. Large dropout rate and small sample size did not permit valid statistical comparison at 6-month follow-up.

Another study (Lee et al., 2010) compared TAU vs. a psychosocial intervention for relapse prevention (PIRP) as add-on to depot antipsychotic (risperidone). The PIRP program consists of psychoeducation for long-acting injections, early detection of warning symptoms, relapse prevention, regular family education, crisis intervention, and encouragement to patients to adhere to a schedule of hospital visits over a 1-year period. Injection frequency was used as a measure for adherence. Results indicated better adherence associated with the intervention as compared to TAU at the end of treatment and 1-year follow-up (*p* < 0.01). Relapse rate at the end of the intervention (*p* < 0.01) and at 1-year follow-up (*p* = 0.04) were significantly lower in the PIRP group compared to the TAU group. Occurrence of injection discontinuation was significantly lower in the PIRP group than in the TAU group. Both groups showed significant improvement in symptom severity, with no difference between the treatment groups.

During a 12-month open-label, randomized controlled trial (Noordraven et al., 2017), patients were allocated to either receive a financial reward on top of usual treatment every time they received their prescribed depot of antipsychotic medication or to receive TAU only, in which patients were encouraged to continue their prescribed depot antipsychotic. Adherence was measured as the number of depots received over the number of prescribed depots during intervention period. Results showed that financial incentives improved LAI adherence significantly better compared to the control group by the end of treatment (33.1%; 95% CI, 20.2–45.4; *p* = 0.031). Also at 6-month follow-up, when financial incentives were discontinued, the positive effects on medication adherence decreased but remained significantly higher in the intervention group than in the control group. However, no differences between the groups were found in symptom severity, hospitalization or hospitalization duration, subjective quality of life, and psychosocial functioning.

#### Technology Interventions

Four domains of technological interventions were identified here: electronic monitoring (Frangou et al., 2005; Velligan et al., 2013), SMS reminders (Montes et al., 2012), a telephone intervention problem solving intervention (Beebe et al., 2017), and the Medication review tool (Moncrieff et al., 2016).

Two studies (Frangou et al., 2005; Velligan et al., 2013) evaluated the effects on adherence of electronic monitoring (e-monitoring) using smart pill containers to a number of different comparators. Results of one study, which randomized patients to receive either Pharm-CAT, Med-eMonitor, or TAU are described above (Velligan et al., 2013). Another study (Frangou et al., 2005) examined how the method of measuring medication adherence (i.e., self-report, pill counting, or e-monitoring) could influence adherence. Results indicated that adherence improved significantly in the e-monitoring group as compared to the control and the pill-counting group. Larger clinical improvement was reported for the e-monitoring group and pill-counting group.

Montes et al. (2012) demonstrated that daily SMS reminders to take medication resulted in better adherence compared with usual care. Greater improvement in clinical symptoms and quality of life at the end of intervention was observed with SMS reminders, but these differences were not preserved at 6-month follow-up. No differences in illness insight were observed between the groups at any measurement points.

Patients in another 6-month study (Beebe et al., 2017) were randomized to receive either telephone intervention problem solving (TIPS), a telephone nursing intervention that is used to provide weekly support to outpatients with PSD (Beebe and Tian, 2004; Beebe, 2005), or TAU. Although pill count adherence did not differ between the groups at the end of the study, significantly more patients in the experimental group had serum antipsychotic levels within therapeutic range.

The Medication Review Tool (Moncrieff et al., 2016), an online form to help patients identify both benefits and issues of their current antipsychotic treatment and any desired changes, had to be taken into psychiatric consultation allowing the patients to express their views more clearly and to have their concerns addressed more systematically about medication. This method improved of adherence in the intervention group compared to controls. Moreover, attitudes toward antipsychotic treatment were also more favorable in the intervention group. No differences in symptomatology and side effects were reported.

## Discussion

### Modifiable and Non-modifiable Risk Factors for Non-adherence

Antipsychotic medication non-adherence is one of the most important challenges that clinicians face in treating psychotic disorders. Subsequently, this review aimed at providing a comprehensive description of the most important factors associated with adherence and the endeavors to improve adherence in this highly prevalent condition. Our results indicate that predictors of medication adherence can be divided in modifiable and non-modifiable risk factors. Non-modifiable risk factors of non-adherence include sociodemographic features, such as younger age (Coldham et al., 2002; Quach et al., 2009; Bayle et al., 2015) and younger age at illness onset (Coldham et al., 2002; Klingberg et al., 2008), and can help to identify at-risk individuals for targeted adherence interventions. Modifiable risk factors, on the other hand, are of particular interest as targets for the development of specific interventions or strategies to improve adherence. Important modifiable risk factors include family and therapeutic relations, as well as some clinical symptoms that may be amenable to treatment. In particular, higher scores on PANSS positive and global psychopathology subscale items [paranoia (Janssen et al., 2006), hostility (Lindenmayer et al., 2009), excitement (Yang et al., 2012), and preoccupation (Yang et al., 2012)] may need to be tackled in order to improve medication non-adherence, although the extent to which positive and negative symptom domains are predictive of adherence behavior remains unclear. Additionally, current but not previous misuse of cannabis represents a clear risk factor for non-adherence, pointing out the importance of abstention strategies toward improving adherence behavior (Coldham et al., 2002; Janssen et al., 2006; Quach et al., 2009; Foglia et al., 2017; Winton-Brown et al., 2017). Unsurprisingly, patients' attitudes and beliefs about medication and illness represent another key modifiable risk factor (Day et al., 2005; Quach et al., 2009; Meier et al., 2010; Yang et al., 2012; Molteni et al., 2014) across all stages of the disorder. A clear positive impact on adherence may be generated by involving family members (Klingberg et al., 2008; Baloush-Kleinman et al., 2011; Winton-Brown et al., 2017) that support the patient and their treatment. Somewhat surprisingly, we found that treatment-related variables, such as administration route, dosage, type of antipsychotic, and medication side effects, do not significantly influence medication adherence. However, study designs may have confounded the results.

### Evidence-Based Strategies to Strengthen Adherence

Despite these important clues, the main drivers and causes of non-adherence in psychosis remain difficult to determine due to the limited quality and heterogeneous nature of the available evidence, leading to a “black box effect,” which has not been very informative for clinicians or researchers. The scarcity of evidence on interventions to improve adherence to antipsychotics stands in sharp contrast with the number of clinical trials trying to prove their effectiveness. Current evidence-based interventions to improve adherence include family therapy, technology-based interventions, and strategies combining depot medication with psychoeducation. However, these findings must be interpreted with caution, given the wide range of heterogeneous interventions, the lack of consequent replication, and methodological restraints. Because of the large influence of patients' attitudes on adherence behavior, naturalistic or non-randomized designs are particularly problematic. There is a need for more well-controlled longitudinal RCTs, assessing both short- and long-term effects on adherence behavior as well as clinical and functional outcome measures. Additionally, rather than studying hybrid interventions consisting of multiple non-specific and partially overlapping components (e.g., CAT, CBT, AT, family therapy), we should study the effectiveness of specific elements of these interventions in tackling one or more of the abovementioned modifiable risk factors, allowing for an adherence strategy that is cost effective and tailor-made to an individual patient. Table 5 outlines our proposed recommendations for such an integrative adherence strategy, based on patients' risk profile. Preventative strategies should be implemented for patients with low-risk profiles, as low vulnerability does not exclude future non-adherence behavior. Assessing patients' adherence behavior (i.e., self-report, family, and interviewer rating) and increasing awareness of their illness and of the benefits of their antipsychotic treatment may reinforce patients to proactively manage their disorder. Patients with a higher vulnerability for non-adherence should be monitored more closely, using both subjective and objective instruments. Where technology-assisted methods are not practical or affordable, prescription refill rates in combination with unexpected pill counts can be performed. Special attention for younger patients is advised. Aside from psychoeducational strategies, above-mentioned evidence-based interventions to improve adherence can be applied to patients at risk of antipsychotic medication non-adherence. Where applicable, patients' family should be involved and educated on this debilitating illness and benefits of a followed treatment course, and cessation of substance use should be encouraged.

**Table 5:** Outline potential adherence strategies.

	**Low risk for non-adherence**	**Vulnerability for non-adherence: 1–2 risk factors**	**High risk for non-adherence: ≥3 risk factors**
Patient profile	Patients with illness insight; positive attitude toward medication; family involvement and support; positive attitudes of family members toward medication	Young patients; patients who lack illness insight, cannabis use and substance dependence; high intensity of symptoms; poor premorbid functioning; negative attitude toward medication; lack of family involvement	
Adherence measurement method	Subjective rating scale	Subjective rating scale + unexpected pill count + prescription renewal/refill	Subjective rating scale + unexpected pill count + prescription renewal/refill + TDM or e-monitoring
Potential intervention strategies	PE	(LAI+) PE + SMS reminders If applicable: family therapy; cessation of cannabis and other substances	LAI + PE + contingency management (incl. financial incentives) If applicable: family therapy; cessation of cannabis and other substances; technology interventions

### Measuring Adherence

A variety of measures of adherence behaviors are available to researchers and clinicians studying populations with psychiatric disorders. However, none of these tools are exact measures of drug intake, and thus, all suffer from limitations. The so-called digital drugs, consisting of an antipsychotic embedded with a sensor to track consumption of the drug, could resolve this issue. However, evidence of better adherence with digital drug is very weak (Cosgrove et al., 2019). Although no gold standard approach to the measurement of adherence exists, some measures are clearly more sensitive and reliable in identifying mismatch between actual and prescribed use of antipsychotics. Measures of medication adherence can be classified in (1) subjective measures of medication use (patient self-report or interviewer ratings) and (2) objective indicators of medication intake, such as pills counts, electronic monitoring, and serum or plasma levels of antipsychotics (see Table 6). Despite the availability of sensitive instruments, no reliable prevalence data on non-adherence in psychosis are available, with reported non-adherence rates ranging from 0.9% (Klingberg et al., 2008) to 81% (Weiden et al., 2012). Shockingly, half of the included studies relied solely on subjective reports or rating scales of adherence. Several studies also used unstandardized and unvalidated subjective adherence measurement tools (Coldham et al., 2002; Bechdolf et al., 2005; Borras et al., 2007; Kahn et al., 2008; Acosta et al., 2009; Mohamed et al., 2009; Quach et al., 2009; Yang et al., 2012; Molteni et al., 2014; Winton-Brown et al., 2017). Valid and reliable therapeutic drug monitoring methods are now increasingly available for the most common antipsychotic drugs (Patteet et al., 2014), and it is hard to understand why TDM is not used more widely, both in clinical practice and in studies that have a primary or secondary focus on adherence assessment. Despite the difficulties linking adherence directly to patients' outcomes, we strongly recommend all clinical trials of treatment interventions for psychotic disorders to routinely include quantifiable and objective measures of adherence rather than only relying on intention-to-treat analysis.

**Table 6:** Advantages and disadvantages of objective and subjective measurement tools.

**Measurement**	**Advantage**	**Disadvantage**
**Objective**		
TDM	- Objective	- Dependent on patient's metabolism - Not quantitative - Does not exclude partial adherence - Cost - Availability
Pill count	- Easy to apply to all patients - Does not require training - Low cost	- Missing data - Reliability
Pharmacy refill, including MPR	- No missing data - Not obtrusive	- Accuracy - Variation in decision rules per study
Monitoring devices (smart containers)	- Reminders - Alert patients if cap is left off of bottle - Notifications of opening cap - Automatic download of data - Multiple drugs with one device	- Leaving caps off of bottle results in missing data in most devices - High cost - Training - Underestimating adherence when multiple pills are taken out at once - Overestimating adherence with multiple openings and no pills have been taken out
**Subjective**		
Self-report and observer-rated	- Easy - Short - Some take time into account - Some Likert-type rating scale	- Cost - Some no specific timeframe - Some dichotomous - Validity - Memory bias - Poor insight may limit accuracy

In addition, given the far-reaching consequences of medication non-adherence in clinical practice, a failure to scientifically address this issue will have important implications for the treatment of patients with psychosis. A proper research agenda to define the optimal treatment of patients suffering from psychotic illness must therefore include the need for a clear definition of adherence, including partial adherence and non-adherence, the need for consensus on appropriate adherence assessment methods, on how to assess individual patients' risk of non-adherence, and which interventions can be applied as part of a personalized and evidence-based treatment plan.

### Limitations and Future Directions

Our review has several limitations. The existing literature is marked by lack of consensus about defining and measuring adherence in PSD, leading to a wide range of adherence rates (0.9–81%) found in the literature. Despite our rigorous inclusion and exclusion criteria, aiming at incorporating only high-quality studies, methodological flaws and heterogeneous definitions, measures, and intervention strategies complicated the quantitative comparison of effects across different studies. In terms of our methodology, while our stringent approach using MeSH terms in our search string improved the quality and specificity (Baumann, 2016) of our literature search, this may have come at the expense of losing some sensitivity to detect all relevant publications. To minimize the risk of missing some relevant studies, we made sure to manually review the reference lists of all individual studies and systematic reviews on the topic. Moreover, our systematic search has been limited to only one search engine. Despite these limitations, we have been able to classify factors associated with antipsychotic medication adherence as modifiable and non-modifiable risk factors to identify possible intervention strategies and to propose evidence-based recommendations.

## Conclusions

One of the greatest problems when dealing with psychotic spectrum diseases is the effectiveness of antipsychotic treatment, which is complicated as patients often fail to adhere to their treatment, adding to the negative effect on prognosis in psychotic illness. Subsequently, this systematic review aims to facilitate endeavors to improve antipsychotic adherence behavior by identifying modifiable and non-modifiable adherence-related risk factors, synthesizing effective intervention strategies, and proposing recommendations to enhance adherence strategies. We demonstrate that non-adherence to antipsychotic medication in patients with psychotic spectrum disorders is a complex process influenced by numerous risk factors, including younger age, poor illness insight, cannabis abuse, and to some extent by present positive symptoms. Positive attitude toward medication, family involvement, and increased insight seem to positively influence adherence. Whereas, several treatment models aimed to improve adherence have been investigated, much ambiguity remains concerning effectiveness and active components. Although much efforts have been invested in investigating adherence, there is a dire need for the implementation of well-validated, standardized assessment methods. To improve long-term outcomes in psychotic patients, we strongly suggest that future treatment strategies should focus on the individual patient's characteristics and needs and the integration of evidence-based interventions into psychiatric services. Such evidence-based integrative treatment strategy is essential in addressing the impact of antipsychotic non-adherence on the patients' prognosis and cognitive and global functioning and on the society.

## Data Availability Statement

The datasets analyzed in this article are not publicly available. Requests to access the datasets should be directed to kawtar.elabdellati@uantwerpen.be.

## Author Contributions

All authors met ICMJE criteria and all those who fulfilled those criteria were listed as authors.

## Conflict of Interest

The authors declare that the research was conducted in the absence of any commercial or financial relationships that could be construed as a potential conflict of interest.

## Abbreviations

AIMS: abnormal involuntary movement scale
AP: antipsychotics
ARS: Adherence Rating Scale
AT: adherence therapy
AP: antipsychotic medication
BIS: Birchwood Insight Scale
BPRS: Brief Psychiatric Rating Scale
CAE: customized adherence enhancement
CAT: cognitive adaptation training
CBT: cognitive behavioral therapy
CDR: concentration to dose ratio
CDSS: Calgary Depression Scale for Schizophrenia
CFI: Camberwell Family Interview
CGI: Clinical Global Impression scale
CRS: Clinician Rating Scale
CVLT, California Verbal Learning Test DAI: drug attitude inventory
DB: double-blind
DKEFS: Delis–Kaplan Executive Function System
EE: expressed emotion
EPS: extrapyramidal symptoms
ES: effect size
ESRS: Extrapyramidal Symptom Rating Scale
FEP: first-episode psychosis
FU: follow-up
GAF: Global Assessment of Functioning
IDS: Inventory of Depressive Symptomatology
LAI: long-acting injectable antipsychotics
LUNSERS: Liverpool University Side Effects Rating Scale
MADRS: Montgomery–Asberg Depression Rating Scale
MAQ: Morisky Green Adherence Questionnaire
MARS: Medication adherence Rating Scale
MeM: Med-eMonitor
MFG: multifamily group therapy
MPR: Medication Possession Ratio
NART: National Adult Reading Test
PANSS: Positive and Negative Syndrome Scale
PE: psychoeducation
PETiT: Personal Evaluation of Transitions in Treatment
PSST: Psychosocial Skills Training
QLS: Quality of Life Scale
QoL: quality of life
RoB: risk of bias
ROMI: Rating of Medication Influences
SAI-C: Schedule for the Assessment of Insight-Compliance
SB: single-blind
SE: side effects
SZ: schizophrenia
SZA: schizoaffective disorder
SPH: schizophreniform disorder
TRQ: Tablet Routines Questionnaire
TAU: treatment-as-usual
WASI: Wechsler Abbreviated Scale of Intelligence
WIS: Wechsler Intelligence Scale
WMS: Wechsler Memory Scale.
